# Plasticity in D1-Like Receptor Expression Is Associated with Different Components of Cognitive Processes

**DOI:** 10.1371/journal.pone.0036484

**Published:** 2012-05-04

**Authors:** Christina Herold, Illah Joshi, Omar Chehadi, Michael Hollmann, Onur Güntürkün

**Affiliations:** 1 Institute for Cognitive Neuroscience, Department of Biopsychology, Faculty of Psychology, Ruhr University Bochum, Bochum, Germany; 2 Department of Biochemistry I—Receptor Biochemistry, Faculty of Chemistry and Biochemistry, Ruhr University Bochum, Bochum, Germany; 3 C. & O. Vogt Institute for Brain Research, Heinrich-Heine-University, Düsseldorf, Germany; Bowling Green State Universtiy, United States of America

## Abstract

Dopamine D1-like receptors consist of D1 (D1A) and D5 (D1B) receptors and play a key role in working memory. However, their possibly differential contribution to working memory is unclear. We combined a working memory training protocol with a stepwise increase of cognitive subcomponents and real-time RT-PCR analysis of dopamine receptor expression in pigeons to identify molecular changes that accompany training of isolated cognitive subfunctions. In birds, the D1-like receptor family is extended and consists of the D1A, D1B, and D1D receptors. Our data show that D1B receptor plasticity follows a training that includes active mental maintenance of information, whereas D1A and D1D receptor plasticity in addition accompanies learning of stimulus-response associations. Plasticity of D1-like receptors plays no role for processes like response selection and stimulus discrimination. None of the tasks altered D2 receptor expression. Our study shows that different cognitive components of working memory training have distinguishable effects on D1-like receptor expression.

## Introduction

The prefrontal cortex (PFC) provides the capacity to interpret and predict incoming information based on past events and to select alternative responses. This capability requires working memory (WM) – a cognitive process in which information is held online and manipulated [1]. One key modulator of WM in the PFC is the neurotransmitter Dopamine (DA) [2]. The tuning of PFC neurons during WM processes and WM performance depend on DA D1-like receptor stimulation [1], [3], [4].

In vertebrates, DA mediates its physiological functions through two pharmacologically and physiologically distinct subfamilies of G protein-coupled receptors, D1-like (D1 and D5) and D2-like (D2, D3, and D4) receptors. The D1-like receptor family is extended in birds, comprising the D1A/D1, D1B/D5, and the D1D receptors, the latter of is physiologically compared to the D1 receptor [5], [6]. DA receptors are differentially expressed in the brain [7]. Both D1 and D5 receptors are coexpressed in prefrontal pyramidal neurons and interneurons, showing a complex pattern of localization at the synapse. [8]–[11]. This difference in subcellular localization suggests that although D1 and D5 receptors exhibit similar pharmacology, they are not functionally redundant. Probably, they are able to complement each other at the behavioral level since D1 receptor knockout mice with intact D5 receptors display normal WM performance, despite showing some learning impairment [12].

Recently, it was shown that cortical D1-like receptor binding changed in association with cognitive training in humans [13]. Further, DA receptors can stimulate their own expression [7]. These results open up the possibility that different cognitive processes induce the expression of different dopamine receptors in various forebrain structures, thereby importantly altering the neurochemical architecture of the cortex. However, several problems have to be solved before such a scenario can be considered likely. First, functions like “working memory” or “cognitive training” involve several subprocesses that reach from the acquisition and retrieval of simple stimulus-response associations to higher cognitive functions. Without separating these components, it remains unclear which function is related to changes in receptor binding. Second, drugs or ligands that specifically affect or bind to D1A, D1B, or D1D are not available, making a classic behavioral or physiological pharmacological approach difficult. Third, different brain structures may show divergent alterations of training-induced changes in receptor binding.

**Figure 1:**
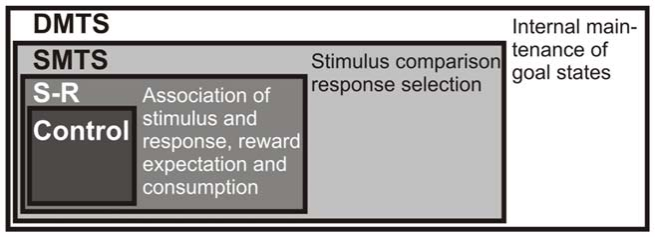
Schematic depiction of the logical structure of the behavioral approach. Expression levels of dopamine receptors are tested in different animal groups under control conditions (no operant behavioral task involved), and during execution of an S-R, an SMTS, or a DMTS task. Much like Russian “Matryoshka” dolls, each of the tasks involves the cognitive components of the previous one, but adds new components that are depicted on the right side of each box.

**Figure 2:**
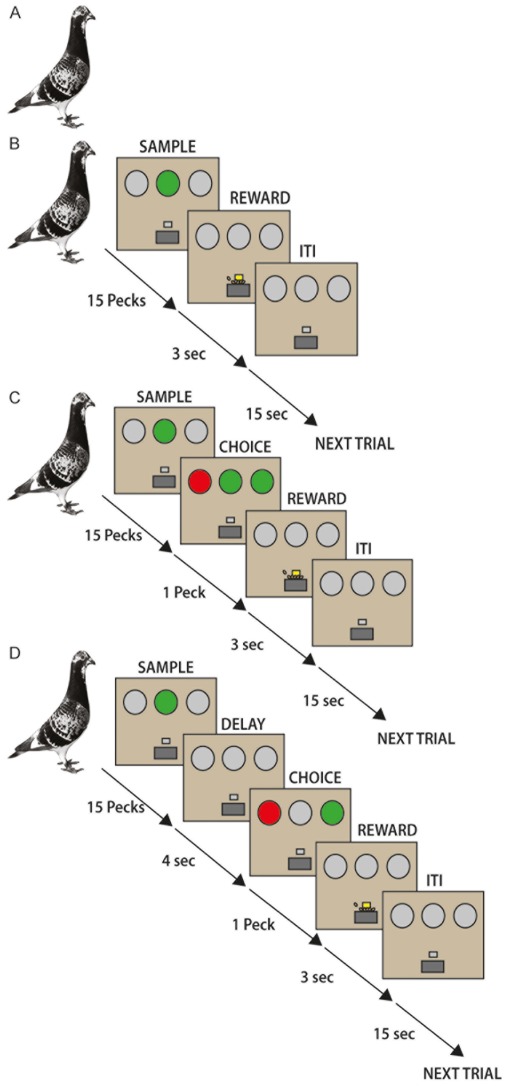
Schematic illustration of the different paradigms for the animal groups in cognitive training. (A) Control group without training in an operant task. (B) S-R task. During training with colored operant keys, each trial started with the presentation of either a green or a red stimulus on one of the three keys. After 15 correct pecks the REWARD phase started with 3 s food access. This was followed by an intertrial interval (ITI) before the next trial started. (C) SMTS task. Training in the simultaneous matching-to-sample task always started with the presentation of either a green or red stimulus as the SAMPLE on the central key. 15 pecks onto this directly started the CHOICE period, where the pigeons had to peck the lateral key that matched the color of the sample. During this phase all keys were simultaneously illuminated. No maintaining of information was required. A single correct peck started the REWARD phase with 3 s food access. This was followed by an ITI before the next trial started. (D) DMTS task. During training of the delayed matching-to-sample task each trial started with the presentation of either a green or red stimulus as the SAMPLE on the central key. 15 pecks onto this started a 4 s DELAY period during which the animals had to memorize the sample color. Then, the lateral keys lit and started the CHOICE period, where the pigeons had to peck the lateral key that matched the color of the sample. A single correct peck started the REWARD phase with 3 s food access. This was followed by an ITI before the next trial started.

We therefore conducted a behavioral working memory paradigm that works like Russian Matryoshka dolls: The four tasks were designed with increasing cognitive demands such that task 2 had one cognitive component more than task 1, task 3 had more components than task 2 and so on (Figure 1 and 2). By subtraction of cognitive faculties between tasks, expression changes of D1A, D1B, and D1D in striatum and avian nidopallium caudolaterale, an avian functional analogue to the prefrontal cortex, could therefore be mapped to specific subcomponents of cognitive training.

## Results

### Analysis of DA Receptors in the Pigeon’s Brain

Prior to testing, we successfully confirmed the presence of the D1A, D1B, D1D, and D2 receptor in pigeons. We isolated parts of the coding DNA sequence for the different receptor genes in the pigeon’s brain after mRNA isolation by using PCR with oligonucleotides that were designed based on the highly homologues sequences for each receptor gene in humans, mice, and chicken. We verified the PCR products by sequence analysis and compared the obtained sequences with sequences in the GenBank® library by using the software tool BLAST®. The derived parts of the coding regions for the different dopamine receptors in the pigeon were deposited in GenBank (EU190460, EU190461, EU190462, EU190463). To analyze the expression of the dopamine receptors after different behavioral training procedures, we redesigned the oligonucleotides based on the pigeon’s sequence to create a subunit-specific set of primers (Table 1). When comparing the obtained cDNA sequences to dopamine receptor sequence in other species, we found that D1A and D1B display substantial similarities to mammalian D1 and D5 receptors, respectively. By contrast, D1D, which is also found in chicken and zebra finch [5], [6], does not have a counterpart in the mammalian brain (Table 2). Recently, the chicken D1D receptor was renamed D1C (gi:118092829 replaced 50749575); however, it is not clear whether this change was based on established similarity to the D1C gene found in other vertebrates. Therefore, we will continue to use the old term D1D. The avian D2 receptor appears to be similar to the mammalian D2 receptor (Table 2).

### Changes in Dopamine Receptor Gene Expression after Prolonged Cognitive Training

The pigeons used for the real-time RT-PCR analysis were age-matched and housed under standard conditions. Pigeons in the control group were inexperienced to any operant or cognitive task, while the pigeons in the experimental groups had learned the described S-R, SMTS, or the DMTS task (Figure 2). Animals in the S-R group were trained in 39±13 sessions, in the SMTS group 40±6, and in the DMTS group 52±26 sessions (all data mean ± SD; F_2.27_ = 1,84, p = 0.18 n.s.). This corresponds to a period of 9±3 (mean ± SD) weeks of training in a specific task. The animals’ forebrains were subsequently extracted and divided into two areas of interest. The first and critical one was the nidopallium caudolaterale (NCL) in the posterior telencephalon (caudal to stereotaxic coordinates A 6.25). As outlined in the discussion, the NCL is a functional analogue to the PFC. The second area of interest consisted of the anterior forebrain frontal to A 8.00. This anterior chunk included a major part of the striatum, although visual and somatosensory areas were also present. Since levels of DA innervation and DA receptor densities are very low in this part of the anterior pallium, the data from the anterior chunk mostly represent striatal DA receptors [14].

RNA was extracted from both areas and a two-step real-time RT-PCR was performed. Data for DA receptor expression levels were normalized to the expression level of the housekeeping gene histone H3.3B from each particular sample of analyzed brain areas and groups.

DA receptor expression levels in the control, the S-R, the SMTS, and the DMTS groups for the NCL and the anterior forebrain (aFB) were analyzed with repeated measurement ANOVAs (4×4×2). Significant main effects for the expression levels of DA receptors were detected between groups (F_3.36_ = 12.14, p<0.001), brain regions (F_1.36_ = 28.04, p<0.001), and DA receptors (F_3.108_ = 55.40, p<0.001). Further interactions were observed between DA receptors and groups (F_9,108_ = 5.09, p = 0.001) as well as brain regions and DA receptors (F_3,108_ = 16.08, p<0.001), and a triple interaction was confirmed between brain regions, DA receptor, and groups (F_9,108_ = 2.28, p = 0.02).

**Figure 3:**
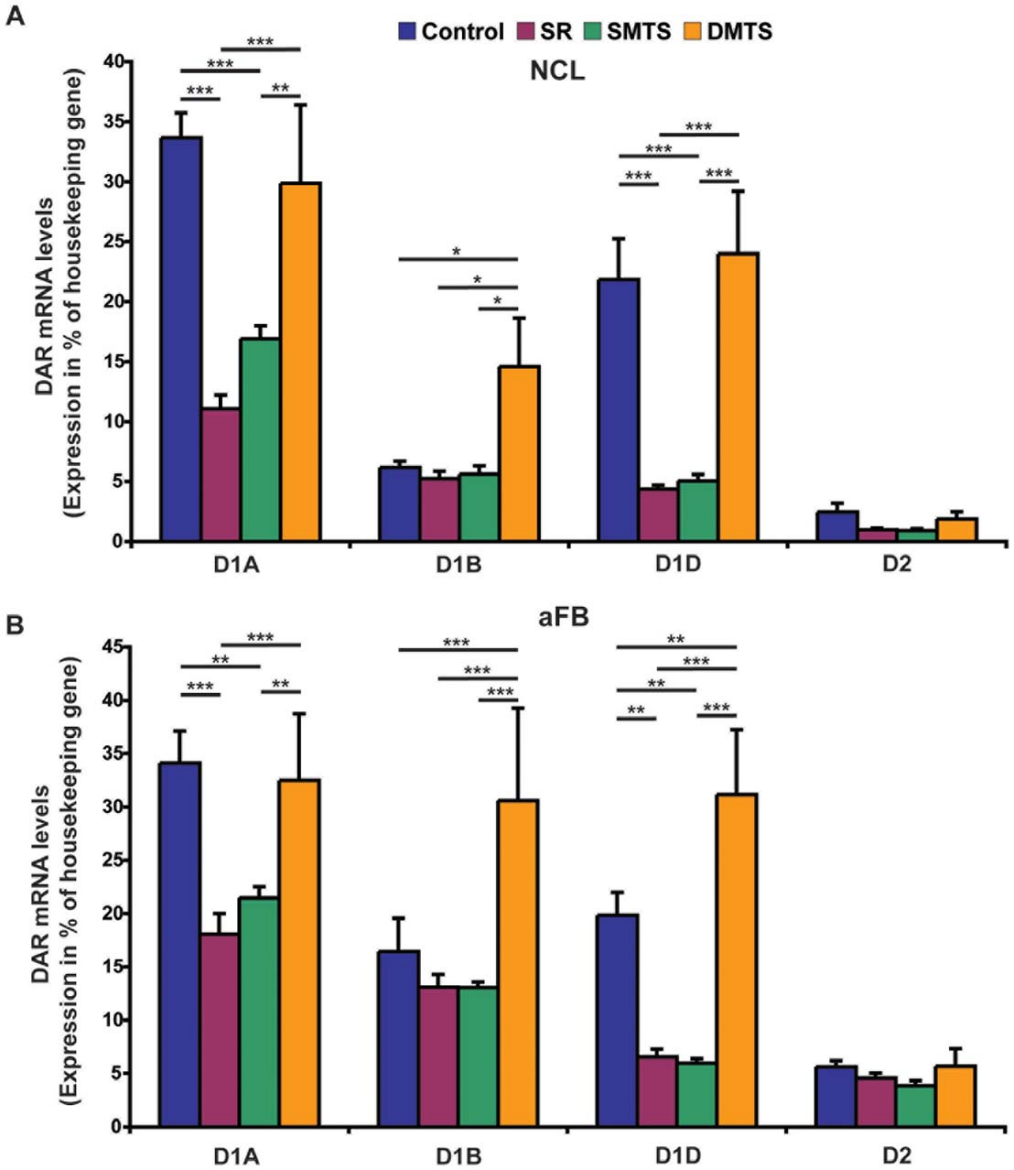
Quantification of dopamine receptor (DAR) mRNA levels in the NCL (A) and the anterior forebrain (aFB; B) of the control and the trained groups. Expression of different DA receptors at the mRNA level is shown relative to the expression of the housekeeping gene histone H3.3B (mean ± SEM; n = 10 each group). Significant differences between groups are marked with asterisks (*p<0.05; **p<0.01; ***p<0.001).

**Figure 4:**
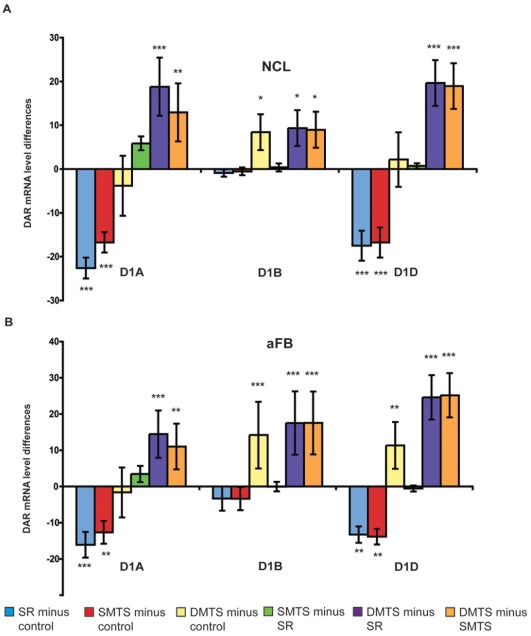
Differences of D1-like mRNA levels in the NCL (A) and the anterior forebrain (aFB; B) between the trained groups. In the NCL and in the aFB, D1A receptor expression levels decreased in the S-R and in the SMTS groups, and increased to control levels after training in the DMTS group. D1B receptor expression increased in both areas in the DMTS group. D1D receptor expression levels decreased in the S-R and the SMTS groups in both areas, and increased to control levels in the NCL while increasing above control levels in the aFB. Thus, a rigid training program that involved a reward-dependent learning of an association between external stimuli and own responses resulted in a down-regulation of the expression of D1A and D1D. D1B expression is only affects after DMTS training. A sole comparison of control and DMTS tasks would have resulted in the wrong conclusion that a DMTS procedure increases D1B expression levels but has no effect on D1A or D1D. All data is presented as mean ± SEM; n = 10 each group. All statistical analyses were only performed on the original data (Figure 3). Significant differences between groups are marked with asterisks (*p<0.05; **p<0.01; ***p<0.001).

Post-hoc analysis revealed that D1A receptor expression decreased in the NCL and the aFB of the S-R and of the SMTS group if compared to the control condition (all p≤0.002, *Fisher-LSD;* Figure 3A and B). Additionally, D1A receptors were expressed at lower levels in the NCL and the aFB of the S-R and the SMTS groups than in the DMTS group (all: p≤0.002, *Fisher-LSD;* Figure 3A and B). Neither in the NCL nor in the aFB did we find differences between the DMTS and the control group. That means D1A receptors were down-regulated after training in the S-R task and in the SMTS task, and up-regulated to control levels after training in the DMTS task. This is illustrated in Figure 4, where the additive logic of our behavioral program was used to calculate differences in receptor expression by subtracting expression levels of different behavioral paradigms. Generally, D1A receptors were expressed equally in both brain regions.

**Table 1:** Primers used for real-time RT-PCR.

Gene	Forward primer 5′–3′	Reverse primer 5′–3′	GenBank accession # for amplicon	Size (bp)
D1A	TTTCCGCAAGGCGTTTTCAAC	TGATCTTTTCCAAAGAAACATCAG	EU190460	304
D1B	CTTCTCCAACCTCCTGGGATG	AGTTATTTTGCCTAGTGAAATCTC	EU190462	276
D1D	TACTGGGCCATCGCCAGCC	TAGGTGATGATCATGATGGGC	EU190461	266
D2	ATGGCTGTGTCCAGGGAAAAA	CCCTGCGCTTCGAGCTGTAGC	EU190463	286
H3.3B	GTGCAGCCATCGGTGCGCT	TGCGAGCCAACTGGATATCT	EU196043	128

The primers were used for quantitative RT-PCR. Each primer pair binds specifically the indicated gene without cross-reactions. The obtained fragments were verified by sequence analysis.

**Table 2:** Comparison of pigeon DA receptor probe sequences to gene sequences in chicken (c) and human (h).

	D1A/D1 gene	D1B/D5 gene	D1D gene	D2 gene
D1A probe	284/305 (93%) (c) 220/304 (72%) (h)			
D1B probe		250/275 (90%) (c) 195/293 (66%) (h)		
D1D probe		97/266 (36%) (c) 71/266 (27%) (h)	219/266 (82%) (c) n.a. (h)	
D2 probe				264/286 (92%) (c) 235/286 (82%) (h)

Data is presented as x/y (%), with x the number of identical bases and y the total length of the fragment followed by the percentage value of sequence identity. Similarities to pigeon sequences differ between chicken and human and are generally larger for chicken sequences. For the D1D probe only low correspondences were detected to the D1B/D5 gene, while high correlations were found with the chicken D1D gene. Empty boxes indicate absence of any significant identities.

In contrast to the D1A receptor, D1B receptor expression in NCL and aFB was higher after prolonged training in the DMTS task when compared to the expression levels of the control, the S-R, and the SMTS groups (all p<0.05, *Fisher-LSD*; Figure 3A and B). No differences in D1B receptor expression levels were seen between the control, the S-R, and the SMTS groups in both brain regions. Thus, only the DMTS training increased D1B receptor levels, while in the other groups levels persisted at control values (Figure 4). Apart from that, under control conditions we found higher expression levels for the D1B receptor in the aFB than in the NCL (p<0.001, *Fisher LSD*).

In the NCL and the aFB, D1D receptor expression levels showed the same pattern as for D1A (Figure 4). Lower mRNA levels in the NCL and the aFB were detected between the control and both, the S-R and the SMTS groups (all p<0.01, *Fisher-LSD*; Figure 3A and B). The same results were observed if the D1D mRNA levels in the NCL and the aFB were compared to the levels of the DMTS group (all p<0.001, *Fisher-LSD*; Figure 3A and B). Further, D1D receptor levels in the aFB were higher in the DMTS if compared to the S-R and the SMTS groups (all p<0.001, *Fisher LSD*; Figure 2B). Thus, D1D receptor expression in the aFB initially decreased after training in the S-R and the SMTS tasks, and then rose again to eventually increase above control levels (Figure 4). D1D receptors were equally expressed in both brain regions.

Expression levels of the D2 receptors were stable under all conditions. None of the training procedures altered D2 receptor expression levels in the two investigated brain regions (Figure 3A and B). Further, no significant difference was detected between the expression levels of D2 receptors in the two regions.

## Discussion

This study reports that training of cognitive subcomponents of a working memory task results in a specific pattern of dopamine (DA) receptor expression changes in the pigeons’ “prefrontal cortex” and anterior forebrain. Our results imply that behavioral procedures that were used in most prior studies involved components that had differentially regulated the expression of D1-like receptors; a fact that was not taken into account before. Additionally, we show that D1A, D1D, and D1B differ considerably in the way their expression patterns change after cognitive training.

The regions of interest are the nidopallium caudolaterale (NCL) and the striatum. The NCL is the functional analogue of the mammalian prefrontal cortex (PFC). Numerous studies have shown that both structures share very similar anatomical [14], [15], neurochemical [16], [17], electrophysiological [18], [19], and functional [20], [21] characteristics. This is especially true for the dopaminergic modulation of ‘prefrontal’ functions in birds and mammals [14], [16], [22]–[24]. Thus, despite the non-homologues character of NCL and PFC, DA systems are converging on these two structures for playing very similar roles. The functional similarities of NCL and PFC possibly result from the fact that the dopaminergic systems that derive from the dopaminergic cell groups in the midbrain are homologues in birds and mammals [5], [25]–[27]. One reason for this might be that the development of these dopaminergic systems is older than the divergence of lines of mammals and birds, although some differences in the divers DA systems between species still exist [25], [26], [28]. The anterior forebrain (aFB) sample of the pigeon encompasses several structures of which the striatum is only one. However, only the striatum has very high levels of DA receptors while the mesopallial and hyperpallial visual and somatosensory areas that are also included show moderate densities [14]. Recently, in situ hybridization studies in the zebra finch and in the chicken brain have shown that the expression of D1-like receptors differs in regions that are included in the aFB sample. For example, D1D receptor transcripts are more prominent in the mesopallial and hyperpallial areas than in the striatal parts, while D1A and D1B receptors showed much more higher densities in the striatum [6], [29]. Further D1B receptors were abundant in the chicken mesopallium [6], [29]. To date, no in situ hybridization data for the expression pattern of DA receptors is available for the pigeon, and even the zebra finch and the chicken showed differences in the expression pattern of DA receptors [6]. Thus, we cannot exclude that non-striatal areas also contributed to our results, and therefore the results for the aFB have to be interpreted with caution. On the other hand, the avian basal ganglia are densely innervated by midbrain dopaminergic fibers [14], [30], [31]. Parallel to the situation in mammals, the striatum in pigeons showed higher DA levels compared to the PFC/NCL and the same differences between striatum and PFC/NCL in release and reuptake mechanisms of DA and its metabolites measured by vivo microdialysis studies [16], [32]. Furthermore, the avian basal ganglia are homologous to their mammalian counterparts [27] and process the same functions as in other vertebrates [33]. Since levels of DA innervation and DA receptor densities in the pigeon seemed to be much lower in the meso- and hyperpallial parts of the aFB sample, we assume that the data from the anterior chunk not entirely but mostly represent striatal DA receptors [14].

Studies in humans show that WM training results in an increase in prefrontal, parietal, and striatal activity [34]. WM training improves intelligence [35], and boosts performance in related but untrained tasks by altering striatal activity [36]. Moreover, it was demonstrated that WM training results in decreased D1-like receptor binding of the ligand [^11^C]SCH23390 in the human prefrontal and parietal cortex, concomitant with an increase in WM capacity [13]. The effect could best be explained by a non-linear, inverted U-function that is typical for the dopaminergic effect on D1-like receptors [3]. Similarly, an excessive expression of prefrontal D1-like receptors was associated with impaired WM performance in schizophrenic patients [37]. However, participants of all of these studies were tested in multiple tasks and in procedures that involve diverse cognitive skills. Furthermore, SCH23390 binds to D1A and D1B [38]. Thus, several independent and partly inversely organized effects could have contributed to these results.

Both, D1A and D1D expression in NCL and striatum was decreased when animals performed an S-R or an SMTS task. Because expression levels after S-R and SMTS training did not differ between each other, SMTS-related cognitive operations like stimulus comparison or response selection had no impact on DA receptor expression levels. A hallmark of reward-related stimulus-response learning is the feedback by DA encoding a reward prediction error signal [39]. After learning, DA neurons of the midbrain show reward-predictive activity in response to stimuli that are associated with variables like reward magnitude and reward probability [40]. Cues associated with food consumption elicit PFC DA efflux as well as retrieval of trial-specific information during an SMTS task [41]–[43]. This is also true for pigeons. An elevation of extracellular DA in the NCL was found after SMTS training [16]. D1-like receptors in the NCL are critically involved in learning new S-R contingencies [21] and stimulus selection [24]. Therefore, S-R and SMTS training presumably had produced an increase of DA release and a concomitant binding to D1A and D1D receptors. Long-term DA influx into the n. accumbens resulted in a down-regulation of D1 receptor expression [44]. Further, physical activity not only increases striatal DA [45] but can suppress striatal D1 receptor mRNA transcripts [46]. Therefore, we assume that the training-elicited down-regulation of D1A and D1D receptors in NCL and striatum result from extended periods of training in which external stimuli had to be associated with own actions, and high performance rates resulted in regular bouts of reward.

No alterations in D2 receptor expression were observed, unlike what was seen after motor learning in the striatum of rats [47]. However, Soiza-Reilly et al. (2004) obtained their results during the ontogenetic development of rats. Thus, the observed changes could be influenced by maturational factors of the dopaminergic system. Recently, it was shown that updating training in humans results in higher DA levels in the striatum that is associated with D2 receptor activity without changing D2 receptor densities [48].

Expression levels of D1A, D1B, and D1D were significantly increased after DMTS training when compared with SMTS (Fig. 4). The difference between DMTS and SMTS is the delay component, which characterizes a DMTS-task. Thus, all D1-like receptors are up-regulated when information has to be maintained in WM, and the animal is being faced with delay periods in which the relevant stimuli are physically absent. During delay periods, a memory trace of the relevant information has to be held active. Some PFC and NCL neurons display sustained activity during delay that could hold a memory trace for a subsequent response or an expected outcome [18], [49]. If this activity within the NCL breaks down, the animal is likely to err [18], [50]. Delay time-specific activations of PFC neurons are modulated by the dopaminergic system via D1-like receptors [1], [3], [51]. Blockade of dopaminergic D1-like receptors in the NCL or the PFC disrupts WM performance [1], [52], [53]. Possibly, DA via D1-like receptors stabilizes active prefrontal neural representations against interfering input by altering ionic and synaptic conductance that enhances spike frequencies of preactivated assemblies [4], [54]. Similar results were reported from the songbird basal ganglia [55]. In addition, in monkeys [2] and pigeons [16] increased DA levels in the PFC and the NCL has been observed in DMTS tasks. Our data indicate that expression levels of all three subclasses of D1-like receptors are up-regulated when being confronted over lengthy periods of time with the task to hold a memory trace active during delay periods. However, because D1A and D1D receptor transcripts are down-regulated prior training of the DMTS- task, it may be necessary to have an optimal range or basis level of D1A and D1D receptors to show an excellent performance in the DMTS task that might be not advantageous for the S-R or the SMTS task. Such a dynamic range in modulation of DA receptor transcripts seemed to be also true in the juvenile zebra finch for different processes during song learning [6]. It is important to note that also- the time to obtain a reward was prolonged in the DMTS task, since the reward always followed the response. Thus, the delay to reward delivery was not equalized between tasks. In principle it is possible that this constitutes a further explanation for the different regulation of DA receptor expression profiles in the DMTS task.

Our inverse experimental approach shows that D1 and D5 receptor expression is variably tuned by different cognitive demands. In mammals D1-like receptors not only have differential intracellular trafficking properties [9], [56] but also different densities in spines, dendrites, somata, and axons [8], [10], [11]. Both, in mammals and birds, D1 receptors are often localized in synaptic triads of pyramidal neurons, where glutamatergic and dopaminergic terminals shape the biophysical properties of individual spines [8], [57], [58]. In mammals, D1 but not D5 receptors form heteromeric assemblies with NMDA receptor subunits by selectively coupling to NR1-1a and NR2A subunits [59]. Indeed, during maintenance periods of DMTS-tasks, forebrain neurons in mammals [60]–[64] and birds [18] show sustained activity that are modulated by D1 receptors by increasing the NMDA receptor-induced EPSCs [4], [52]. Our findings of task-dependent altered D1-like expression could imply that these molecular dynamics affect the synaptic surrounding of spines.

By contrast, D5 receptors are predominantly localized within dendritic shafts, where inhibitory GABAergic neurons form postsynaptic contacts [8], [10]. D5 receptors couple through binding to the GABA_A_ receptor γ2 subunit [65], [66]. This D5-GABA_A_ receptor cross-talk allows induction of reciprocal inhibitory interactions. As we found training-induced increased levels of D5 receptor mRNA in the avian forebrain, this opens the possibility of an increased D5 receptor cross-talk with GABA_A_ receptors. Indeed, an increased overall activity of the PFC after cognitive training was reported [34]. Our results support the idea that, at least in birds, D1 and D5 receptors serve distinct cognitive functions and presumably mediate different effects at the cellular level.

## Materials and Methods

### Animals

40 experimentally naive, adult, unsexed pigeons (*Columba livia*) of local stock, where they live in a natural environment, were used in the experiments. For each group ten pigeons were used. All pigeons were age-matched between one and five years. Animals in the control group (Figure 2A) were experimentally inexperienced, while the rest of the pigeons participated in the cognitive training. Dependent on the task they were trained they were divided into the S-R group, the simultaneous matching-to-sample (SMTS) group, and the delayed matching-to-sample (DMTS) group. All pigeons were housed in individual cages in a temperature-controlled room on a 12-hr light-dark cycle. One week before the experiment started, pigeons from all groups were food-deprived to 80% of their normal free feeding weights. They always had *ad libitum* access to water and grit. Thereafter, pigeons of local stock for the control group were directly used for brain tissue preparation. Pigeons participating in the cognitive training were trained and tested four to five days a week in an operant chamber.

### Ethics Statement

The animal procedures were conducted in accordance with the NIH *Guide for the Care and Use of Laboratory Animals* and under adherence to the German laws to protect animals, and hence, the European Communities Council Directive of 18 June 2007. The experimental protocol was approved by national authorities and the ethics committee of the *Landesamt für Natur, Umwelt und Verbraucherschutz* (LANUV) of North Rhine-Westphalia, Germany.

### Apparatus and Stimuli

Two operant chambers (34×33×36 cm) were used in the cognitive training. Each chamber was controlled via a digital input-output board (CIO-PDISO8; Computer Boards, Inc.) and illuminated by a 24 W, centrally fixed light bulb. Three opaque operant keys (2 cm in diameter) with a distance of 10 cm between them were located at the back panel of each box, 22 cm above the floor. The pecking keys were homogeneously transilluminated either by white, red, or green light, without matching the brightness of the colors. White lights were used in the operant conditioning and pretraining sessions, while red and green lights were used during the pick training, the SMTS, and the DMTS tasks. The feeder, combined with a light-emitting diode, was fixed in the center of the back panel, 5 cm above the floor.

### Behavioral Procedures

The logical structure of the behavioral approach is depicted in Figure 1 and Figure 2.

#### Pretraining

During the first sessions pigeons were trained to peck reliably on the center key, whenever it was illuminated with white light. After a single peck, the light was turned off, and the pigeons were reinforced with 3 s access to food, followed by an inter-trial interval of 5 s. In the next steps each trial began with the illumination of the center key. One peck on the lateral keys during this phase terminated the trial that was then followed by an inter-trial interval of 15 s and a retry of the trial. Pecking on the central key led to the extinction of the central light and, immediately thereafter, to the illumination of one of the lateral choice keys. After pecking the illuminated lateral key, pigeons were reinforced, whereas pecking the dark choice key caused punishment by a 10 s time-out period during which all lights were turned off. One session included 80 trials with a 15 s inter-trial interval between each trial. Throughout the next training sessions, the number of pecks required on the center key to extinguish the center light and to turn on the lateral lights was constantly increased from 1, 3, 6 to 15 pecks. The criterion for the pretraining was 100% correct responses in one session.

#### The S-R task

After pretraining, pigeons were trained for a simple stimulus-response (S-R) task. For this, they learned to peck reliably on one of the keys, whenever it was illuminated with colored light. No discrimination of colors was involved. After 15 pecks, the light was turned off, and the pigeons were reinforced with 3 s access to food, followed by an inter-trial interval of 5 s. Illumination of the either one of the lateral keys or the central key was randomized to exclude a spatial bias for one of the keys. Pecking one of the dark keys caused punishment by a 10 s time-out period during which all lights were turned off. One session included 80 trials with a 15 s inter-trial interval between each trial. Before decapitating the animals for quantification of the different dopamine receptor subtype mRNA levels in the nidopallium caudolaterale (NCL) and the anterior part of the forebrain (aFB), all pigeons had to reach an overall criterion of 80% correct responses on three subsequent days. Taken together, the S-R task demanded of the animals to track the location of the colored key and to repeatedly peck it to then obtain reward (Figure 2B).

#### SMTS task

After pretraining, the operant keys were illuminated with colored light. The illumination of the central stimulus with either red or green light started the trial. The center light stayed on until the pigeon had pecked the key 15 times. Immediately thereafter, the two lateral choice keys were illuminated simultaneously, one in red and the other in green light, while the central key stayed on. Pigeons were reinforced after pecking the lateral illuminated choice key that matched the color of the simultaneously illuminated central key with 3 access to food, and were punished after pecking the non-matching key by a 10 s time-out. No maintaining of stimulus information was required to perform the task because during the choice phase all keys were illuminated. Training went on until the pigeons reached a performance level of 80% correct responses on three subsequent days. The order in which colors were presented was randomized, so that pigeons could not learn a fixed sequence of presentation of the stimuli. Taken together, the SMTS task demanded of the animals to do the very same as in the S-R task until the 15^th^ peck on the central key. However, immediately thereafter they had to match the color of the central key to one of the choice keys and then to select a response to this identified key (Figure 2C). Thus, relative to the S-R animals, the SMTS group had to additionally perform a color matching and response selection task component.

#### DMTS task

To introduce WM with a short-term memory component, we used a DMTS task. Each trial began with the illumination of the central key, the sample stimulus, either in red or green. During this time, pecking on the lateral dark keys terminated the trial and an inter-trial interval was initiated followed by a repetition of the trial. Otherwise the sample stimulus remained active until the pigeon had pecked the sample stimulus 15 times. After that the delay period started during which the sample stimulus was no longer visible. At the end of the delay the two lateral choice keys were illuminated simultaneously, one in red and the other in green light. Matching the sample stimulus by choosing the choice key with the same color as the sample stimulus before (correct response) was rewarded immediately with free access to food for 3 s. Choosing the complementary color which was not shown at the previous sample stimulus (incorrect answer), was punished with a 10 s time-out period in darkness. The next trial started after a 15 s inter-trial interval. Each session consisted of 80 trials. The order in which colors were presented was randomized, so that pigeons could not learn a fixed sequence of presentation of the stimuli (Figure 2D).

Pigeons of the DMTS group were first trained on a 0 s delay task until they reached a performance level of 80% correct matches in at least three subsequent sessions. Afterwards the delay level was augmented from 0 to 1 s until they reached criterion after which the delay was increased again to 2 s, and later up to a maximum of 4 s. Pigeons had to reach an overall criterion of 80% correct responses on the maximum 4 s delay in at least three subsequent sessions before they were decapitated for the quantification of the different DA receptor subtype mRNAs. Thus, relative to the SMTS animals, the DMTS group had to additionally maintain color information in working memory during the delay period.

### RNA Preparation and Quantitative Real-time RT-PCR

For brain tissue preparation pigeons were deeply anesthetized with Equithesin (0.5 ml/100 g body weight, i.m.) and decapitated. Brains were quickly removed and stored on ice. The NCL and the anterior parts of the forebrain including the striatum were dissected out for the left and right hemispheres separately, frozen in liquid nitrogen and stored at −80°C for later use. First, the pigeon brain was adjusted under a binocular microscope with a µm scale. Second, the anterior chunk of the forebrain (aFB) frontal to A 8.00 was cut off straightly from each brain half. Herein, the cerebellar-forebrain junction was used as a reference point and additionally the length of the forebrain itself. According to the atlas of Karten and Hodos [67] these sample included a major part of the basal ganglia as well as visual and somatosensory areas like the entopallium and the frontal parts of the meso-, hyper- and nidopallial regions. Third, the NCL sample according to Waldmann and Güntürkün [68] was prepared (For a detailed atlas of the NCL see [17]). Because a large part of the half-moon-shaped NCL starts caudal from the stereotactic coordinate A 6.25, we cut off a further slice with a thickness of 2 mm to achieve A 6.00. After that we removed the ventrally positioned arcopallial parts. We used the tractus dorso-arcopallialis to orientate because this tract is highly visible in the native preparation. In the next step we cut off the medial parts of the nidopallium, namely the nidopallium caudolaterale central and the nidopallium caudo mediale as well as the hippocampal and the overlaying CDL regions that are naturally separated from the NCL by the ventricle. Therefore, this sample consists mostly of NCL material. Total RNA was extracted to process for real-time RT-PCR by using the NucleoSpin®RNA II Kit (Macherey-Nagel, Düren, Germany). RNA quality was checked for each probe. cDNA was obtained with the Superscript™II RT First Strand Synthesis System for RT-PCR (Invitrogen, Karlsruhe, Germany). For each probe 300 ng of total RNA was used for the RT reaction. Each probe was replicated twice.

Real-time PCR was performed on a LightCycler® (Roche, Mannheim, Germany) to determine the mRNA expression in the NCL or the anterior parts of the forebrain. For the preparation of the PCR standard reaction the protocol from LightCycler® FastStart DNA Master^PLUS^ SYBR Green I (Roche, Mannheim, Germany) at a total volume of 20 µl was used. For each sample 1 µl cDNA diluted with 4 µl PCR-grade water was used as template for the reaction, with 10 µM forward and backward primers. Both, targets and reference amplifications were performed in triplicate in separate capillaries. The primers for the different DA receptors and the housekeeping gene histone H3.3B used in the real-time PCR are listed in Table 1. Thermal cycling conditions included 10 min at 95°C preincubation, followed by 40 amplification cycles comprising 95°C for 10 s, 60°C for 10 s, and 72°C for 20 s, and one cycle for melting curve analysis comprising 95°C for 0 s, 65°C for 15 s, and 95°C with a slope of 0.1°C/s, followed by cool-down to at least 40°C. Under these conditions the efficiency for all primers was in the range of 2 and thus at maximum. Further, expression of the reference gene was controlled in all groups. None of the groups showed regulation in H3.3B expression.

Real-time PCR products were verified by melting curve analysis, 2% agarose gel electrophoresis (ethidium bromide staining), and sequence analysis on an ABI PRISM Genetic Analyzer 3100C (Applied Biosystems, Darmstadt, Germany). Sequence identities of PCR products to homologues in chicken and human are listed in Table 2.

### Data Analysis

Behavioral data was analyzed with a one-way ANOVA with group as “between subject” factor and training days as “within subject” factor. For analysis of real-time RT-PCR data the levels of target gene expression were normalized to the levels of the housekeeping gene histone H3.3B. Ratios between different groups were calculated with the 2-ΔΔCT method. For statistical analysis of real-time RT-PCR data, all values for the different DA receptor types given as percent expression relative to the housekeeping gene were analyzed between groups with repeated measurement ANOVAs (4×4×2). Therein, group was defined as “between subject” factor, and receptors (D1A, D1B, D1D, D2) and brain regions (NCL and anterior forebrain) were defined as “within subject” factors. If main or interaction effects were confirmed this was followed by post-hoc analysis with *Fisher LSD* tests using Statistica 9 (StatSoft, Tulsa, USA). For all analyses the p-level was set at 0.05.
